# From checkups to change: Longitudinal changes in lifestyle-related factors following repeated occupational health assessments among 106 005 Swedish workers

**DOI:** 10.5271/sjweh.4256

**Published:** 2026-01-01

**Authors:** Daniel Väisänen, Elin Ekblom-Bak, Linnea Eriksson, Lena V Kallings, Magnus Svartengren, Robert Lundmark, Magnus Lindwall, Victoria Blom, Andreas Stenling

**Affiliations:** 1 Department of Physical Activity and Health, The Swedish School of Sport and Health Sciences, Stockholm, Sweden.; 2 Department of Psychology, University of Gothenburg, Gothenburg, Sweden.; 3 Department of Psychology, Umeå University, Umeå, Sweden.; 4 Department of Sport Science and Physical Education, University of Agder, Kristiansand, Norway.; 5 Department of Medical Sciences, Occupational and Environmental Medicine, Uppsala University, Uppsala, Sweden.; 6 Department of Occupational and Environmental Medicine, Uppsala University Hospital, Uppsala, Sweden.; 7 Department of Health, Education and Technology, Luleå University of Technology, Luleå, Sweden.

**Keywords:** exercise, health appraisal, health profile assessment, perceived health, weight

## Abstract

**Objectives:**

We investigated changes in weight, exercise frequency, and perceived health from the first to last health profile assessment (HPA) and between the number of tests within five years. We examined whether sociodemographic factors, or baseline values influenced these changes.

**Methods:**

Data from 106 005 employees with ≥2 HPA (1990–2021) were included. Change between the first and last HPA within a five-year period was analyzed. Baseline age, sex, education, occupation, and baseline values of each outcome were included as predictors. XGBoost models assessed changes in the outcomes, and performance was evaluated via root mean squared error, mean absolute error, and R-squared. We employed Shapley Additive Explanations and forward marginal effects to interpret dose–response relationships and subgroup differences.

**Results:**

Predictive performance was low, suggesting that the included variables only partially explained observed changes. Nonetheless, longer intervals between the first and last HPA correlated with greater weight gain, while a higher number of tests predicted slightly lower weight gain and modest improvements in perceived health and exercise frequency compared to the average change. Younger participants had larger weight increases, whereas those with higher education showed smaller declines in exercise frequency.

**Conclusions:**

Infrequent HPA alone did not appear to substantially influence the lifestyle-related factors studied. However, more frequent HPA, coupled with enhanced feedback and support, may yield small improvements in weight, perceived health, and exercise frequency compared to the average change.

Several non-communicable diseases are, to some extent, preventable with improved health behaviors (1, 2). The workplace is a setting where health promotion activities—focusing on health behaviors and other health-related factors—can be implemented to reach individuals from diverse sociodemographic backgrounds. However, when investigating the effectiveness of health-promoting initiatives to improve healthy behaviors in occupational settings, the literature shows varied results. Two systematic reviews showed small effects on weight (3, 4). Another systematic review and meta-analysis showed that workplace wellness programs can improve diet, waist circumference, and cholesterol levels (5). Other reviews show little-to-no effect of health promotion initiatives on health behaviors, body mass index (BMI), and self-perceived health (6–8).

One common type of health-promoting activity is health assessments. Health assessments include risk assessment on health status, with or without additional feedback or health-promoting activity (9). Several factors may influence changes in health status after a health assessment. A small number of studies have shown a dose–response relationship between the frequency of health assessments or other health-promoting activities and changes in health parameters (10, 11). Additionally, greater improvements in health have been shown for individuals with higher BMI and cardiovascular disease risk at baseline (12). However, reviews investigating differences in the effectiveness of health promotion initiatives in the workplace did not show differences between individuals of different socioeconomic positions (6, 7). Another review focusing on individuals in low socioeconomic positions showed no difference in the results depending on age, gender, or marital status (8). A better understanding of how changes in health parameters may depend on the frequency of activity and individual characteristics could lead to more efficient implementation of interventions, including health assessments in the workplace.

In Sweden, a large-scale health assessment, the so-called 'health profile assessment' (HPA), is available for employees at workplaces connected to occupational health services. HPA is one of the largest ongoing data collections on health assessments that can be used for research. The HPA has been running since the late 1970s, with around 800 000 tests registered in a database since the 1990s, and includes a health assessment with individually tailored feedback. A few smaller studies have investigated changes in health status after performing an HPA. One study showed improvements in cardiovascular fitness and living habits but did not find significant changes compared to a health assessment without individually tailored feedback (13). Another study did not report any results on changes in health status because the attrition was too extensive to draw any conclusions on the effects of HPA (14). No large-scale evaluation of changes in different health parameters after an HPA has been conducted. Therefore, investigating changes in lifestyle- or health-related factors in a larger sample after participating in an HPA would provide important information for employers, occupational health services, and yield implications for public health.

## Research questions

The main aim of the current study is to investigate changes in three lifestyle-related variables (weight, exercise frequency, perceived health) from the first to the last performed HPA in relation to both time between tests and number of tests within five years, and whether changes vary as a function of sociodemographic factors or baseline levels of the outcome variables. Specific research questions (RQ) are: (i) How do lifestyle-related variables change between the first to last test within a follow-up period of a maximum 5 years? Does the time between the first and last test impact change? (RQ1), (ii) Does the number of tests between the first to last test influence change? (RQ2), (iii) Do these changes vary in relation to sociodemographic factors (sex, age, length of education, occupation) and baseline levels of the outcome variables? (RQ3).

## Methods

### Study design and participants

This study utilized data from HPA conducted by occupational health services (OHS) across Sweden. To contextualize the selection of HPA participants, it is important to outline the typical decision-making process enabling an individual to participate in an HPA as previously described (15). Briefly, the process is initiated by the employer, who decides to offer an HPA to employees. A contract is then established with a health assessment provider, most often an OHS. If the chosen provider includes HPA among its services – which is the case for roughly 90% of occupational health services in Sweden – the employer and provider may agree to carry out the HPA. Employees are subsequently informed about the opportunity and invited to participate. Participation is voluntary and free of charge for the employee.

The present study included data from individuals who performed an HPA at two time points or more between 1990 and 2021. Data was chosen according to the flow-chart in the supplementary material (www.sjweh.fi/article/4256) figure S1. Participants with annual weight changes exceeding ±20 kg were excluded as extreme outliers, likely representing measurement errors or underlying medical conditions, given that typical adult weight change is 0.5–1.0 kg per year and an intensive weight loss regime typically achieves 5–8.5 kg loss in six months (16, 17). The final data consisted of 106 005 individuals who had performed 239 103 HPA. Data were aggregated into one row per individual (106 005 rows), showing the change between their first and last HPA in the primary outcome variables of body weight, perceived health, and exercise frequency.

### Health profile assessment (HPA)

The HPA includes three main components (i): a standardized questionnaire on for example physical activity, lifestyle habits, perceived health, and symptoms (ii); a physical examination measuring for example body mass, height, blood pressure, and cardiorespiratory fitness; and (iii) a concluding 20–40-minute behavior change dialogue with an HPA coach using a motivational interviewing approach. Upon completion, data are stored in a centralized database managed by the HPI Health Profile Institute (a private company), which has developed and standardized the method since its inception. Standardization includes a detailed protocol, a 3–4-day training program, and certification of HPA coaches (usually nurses or physiotherapists in OHS). The database supports comparison with normative values, monitoring over time, and workplace-level reporting, while participants can access and benchmark their own results.

### Outcome variables

Weight (in kilograms) was measured in light weighing clothing at both the first and last assessment. The difference between these two measurements (last minus first) was calculated to create a change variable, with higher values indicating an increase in weight over time. Self-rated perceived overall health was assessed using the statement “*I perceive my physical and mental health as…”* with the alternatives *Very poor, Poor, Neither good nor bad, Good, Very good*. Exercise frequency was self-reported using the statement “*I exercise for the purpose of maintaining/improving my physical fitness, health, and well-being…”* with the alternatives *Never, Sometimes, 1–2 times/week, 3–5 times/week,* ≥*6 times/week*. To capture changes over time for these variables, the score at the first test was subtracted from the score at the last test, resulting in a possible range from -4 to 4. Baseline perceived health and exercise frequency were aggregated into three groups for the forward marginal effects analyses by combining the two lowest and two highest categories, respectively.

### Predictors

Predictors included age at baseline, sex, education, occupation, number of performed HPA, and time interval between first and last HPA. The highest level of education was originally assessed on a seven-point scale and aggregated into three categories: primary (≤9 years), secondary (10–12 years), and tertiary (>12 years). Occupational group was determined using the Swedish Standard Classification of Occupations (SSYK). For this study, we aggregated the four-digit SSYK codes into four main categories: blue-collar low-skilled (major level codes: 8–9), blue-collar high-skilled (codes 6–7), white-collar low-skilled (codes 4–5), and white-collar high-skilled (codes 1–3). The number of performed HPA was calculated as outlined in supplementary figure S1 (flow chart) and quantified the amount of HPA received within five years. The time between the first and last HPA was measured in days and converted into years. Baseline values of weight, health, and exercise change were included in their respective models.

### Statistical analysis

Given the exploratory nature of the analyses, machine learning models capable of capturing complex, non-linear relationships and interactions within the data were used, specifically, eXtreme Gradient Boosting (XGBoost) regression models were used to examine the relationship between predictors and changes in the outcome variables (18). For this, the dataset was randomly divided into a training set (80%) and a testing set (20%). The change in the three primary outcomes were evaluated in each XGBoost model as change between first to last HPA, with the predictors time between first and last HPA, number of HPA between first and last HPA, sex, baseline age, education level, occupation, and baseline value of the outcomes.

### Model tuning and evaluation

Hyperparameter combinations were generated using Latin hypercube sampling to efficiently explore the parameter space (19). Parameters included: maximum tree depth, minimum child weight, gamma, subsample ratio, feature sampling ratio, and learning rate. The best model was selected based on the lowest root mean squared error (RMSE) averaged across validation folds, then evaluated on held-out test data.

### Performance metrics

Model performance was assessed using RMSE, R-squared (R^2^), and mean absolute error (MAE). All results presented use test data for unbiased assessment.

### Model interpretation and inference

Shapley Additive Explanations (SHAP) values were employed to quantify variable contributions (20). Essentially, SHAP values quantify how much each variable pushes the prediction away from the baseline. The Tree SHAP method was used for computational efficiency (21). SHAP values were averaged per time point and test occasion, visualized as line plots with 50% and 75% distribution ranges. Variable importance was evaluated using aggregated absolute SHAP values, with bee plots showing directional impacts.

In addition to SHAP, forward marginal effects (FME) were used as a unified, model-agnostic interpretation method for non-linear prediction functions (22). FME attribute to each predictor the exact change in the predicted outcome resulting from a specified change in variable values, considering both univariate and multivariate variable interactions. Conditional average marginal effects (AME) were computed for sex, age, occupation, education, test count, and baseline values, representing the estimated change per three-month increase in time between assessments. Confidence intervals (CI) were calculated using the *t*-statistic method (22).

## Results

A total of 106 005 individuals were included in this analysis. The baseline sample had a mean age of 43.8 [standard deviation (SD) 11.2] years, with 47% identifying as women. Of the total sample, 43% had tertiary education, 53% belonged to white-collar high-skilled occupational groups, and 14% in blue-collar low-skilled occupational groups.

Between the first and last HPA, the median time interval was 2.0 [interquartile range (IQR) 1.0–3.2] years, with a mean of 2.3 (SD 0.6) HPA performed. The descriptive average weight increase for the full sample was 0.49 (SD 4.26) kg, while increases in perceived health and exercise frequency had mean scores of 0.06 (SD 0.74) and 0.15 (SD 1.04), respectively. Further, the yearly weight increase was 0.12 (SD 3.04), perceived health increase 0.10 (SD 0.86), and exercise increase 0.16 (SD 1.21) See table 1 for values per count of HPA and supplementary table S1 for HPA characteristics within 1–5 years from baseline.

**Table 1:** Characteristics of the study population in relation to number of follow-up tests. [SD=standard deviation].

		2 Tests (N=84 207)			3 Tests (N=17 490)			4 Tests (N=3 449)			5 Tests (N=731)			6 Tests (N=128)			Overall (N=106 005)	
		N (%)	Mean (SD)		N (%)	Mean (SD)		N (%)	Mean (SD)		N (%)	Mean (SD)		N (%)	Mean (SD)		N (%)	Mean (SD)
Sex																		
	Men	43 786 (52)			9 838 (56)			1 990 (58)			443 (61)			84 (66)			56 141 (53)	
	Women	40 421 (48)			7 652 (44)			1 459 (42)			288 (39)			44 (34)			49 864 (47)	
Age at baseline (years)			43.7 (11.3)			43.7 (11.2)			44.6 (10.9)			44.9 (11.3)			45.9 (11.5)			43.8 (11.2)
Time from baseline (years)																		
	Within year 1	25 731 (31)			1 406 (8.0)			50 (1.4)			0 (0)			0 (0)			27 187 (26)	
	Within year 2	22 082 (26)			2 603 (15)			333 (9.7)			12 (1.6)			2 (1.6)			25 032 (24)	
	Within year 3	17 842 (21)			3 439 (20)			694 (20)			89 (12)			3 (2.3)			22 067 (21)	
	Within year 4	12 152 (14)			5 019 (29)			1 133 (33)			221 (30)			25 (20)			18 550 (17)	
	Within year 5r	6 400 (7.6)			5 023 (29)			1 239 (36)			409 (56)			98 (77)			13 169 (12)	
Weight at baseline (kg)			78.4 (15.7)			79.0 (15.8)			79.4 (15.8)			80.1 (15.4)			80.6 (13.8)			78.5 (15.7)
Weight change (kg)			0.4 (4.1)			0.7 (4.8)			0.7 (4.9)			0.9 (5.1)			0.8 (5.4)			0.5 (4.3)
Exercise at baseline																		
	Never	8 979 (11)			1 960 (11)			378 (11)			75 (10)			9 (7.0)			11 401 (11)	
	Sometimes	20 200 (24)			4 248 (24)			811 (24)			185 (25)			29 (23)			25 473 (24)	
	1–2 times/week	28 094 (33)			5 940 (34)			1 240 (36)			253 (35)			49 (38)			35 576 (34)	
	3–5 times/week	23 017 (27)			4 567 (26)			866 (25)			187 (26)			36 (28)			28 673 (27)	
	≥ 6 times/week	3 917 (4.7)			775 (4.4)			154 (4.5)			31 (4.2)			5 (3.9)			4 882 (4.6)	
Exercise change			0.1 (1.0)			0.2 (1.1)			0.2 (1.1)			0.3 (1.1)			0.2 (1.2)			0.2 (1.0)
Health at baseline																		
	Very poor	471 (0.6)			83 (0.5)			15 (0.4)			2 (0.3)			0 (0)			571 (0.5)	
	Poor	4 784 (5.7)			925 (5.3)			182 (5.3)			39 (5.3)			3 (2.3)			5 933 (5.6)	
	Neither good or bad	21 833 (26)			4 596 (26)			957 (28)			187 (26)			36 (28)			27 609 (26)	
	Good	47 862 (57)			9 946 (57)			1 928 (56)			435 (60)			71 (55)			60 242 (57)	
	Very good	9 257 (11)			1 940 (11)			367 (11)			68 (9.3)			18 (14)			11 650 (11)	
Health change			0.1 (0.7)			0.1 (0.8)			0.1 (0.8)			0.1 (0.7)			0.0 (0.8)			0.1 (0.7)

For weight change, the final XGBoost model had an RMSE of 4.24 and an MAE of 2.85, resulting in an R^2^ of 3.76% on the test set. Lower RMSE and higher R^2^ were observed for changes in perceived health (RMSE 0.63, R^2^=26.14%) and exercise frequency (RMSE 0.88, R^2^=27.10%). Performance metrics are summarized in supplementary table S2. Metrics from cross-validation and test data were closely aligned, suggesting minimal overfitting and reasonable model generalizability.

SHAP importance values (supplementary figure S2) revealed that, for all three outcomes—weight change, perceived health change, and exercise frequency change—their respective baseline values were the most influential predictors. For weight change, the second and third most important predictors were time between HPA and baseline age. In contrast, perceived health change showed a marked gap in importance after its baseline value, with time between HPA and occupation emerging as the second and third most important variables. Similarly, exercise frequency change displayed a substantial drop in importance after its baseline level, followed by time between HPA and sex.

**Figure 1:**
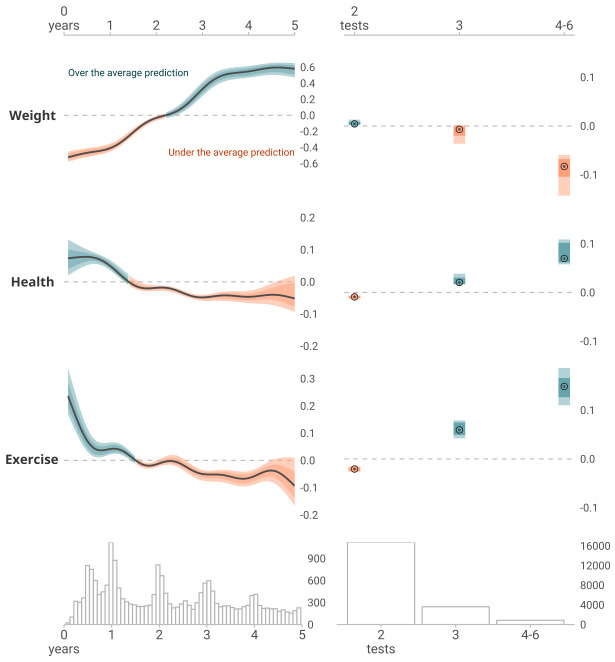
SHAP dependency plots illustrate the impact of time between the baseline and last HPA (LEFT panel) and the number of HPAs between the first and last HPA (RIGHT panel) on model predictions for changes in body weight, perceived overall health, and exercise frequency. In the left panel, the plots display the estimated conditional quantiles (12.5^th^, 25^th^, 50^th^, 75^th^, and 87.5^th^ percentiles) of the SHAP values as a function of time for each outcome. The median line (solid gray/black) and the shaded ribbons representing the interquartile ranges (12.5–87.5^th^ percentile and 25–75^th^ percentile) are derived from quantile generalized additive models, ensuring a smooth and coherent representation of the data. In the right panel, the plots show the SHAP values as a function of the number of HPA. The shaded regions represent the variability in the SHAP values across quantiles (12.5^th^, 25^th^, 50^th^, 75^th^, and 87.5^th^ percentiles). In both panels, the data are compared to the average prediction (the expected prediction) with the dashed gray line representing the global average over the x-axis variable. The color scheme indicates the direction of the variable contribution: blue regions denote instances where the variable contributes to predictions over the average/expected (positive SHAP values), while orange regions indicate contributions under the average/expected (negative SHAP values). Shaded regions and bands illustrate the 50% and 75% intervals of SHAP values, offering a visual representation of the variability in the predictions. For changes in body weight, the SHAP values are interpreted in terms of change in kilograms. For perceived health and exercise frequency, the SHAP values are interpreted on a scale of –4–4, consistent with the ordinal scale used in the self-reported questionnaire during the HPAs. The model consists of the outcome variables, sex, age at baseline, education, occupation and baseline value of the outcome variables. Below both panels are counts for each of the x-axis variables.

Figure 1 illustrates the overall trends for weight change, perceived health change, and exercise change over time and by the number of performed HPA, as estimated by the models. According to the model outputs, the difference in mean SHAP value for weight change (in kg) from year 1 to 5 was an increase of 0.98, for perceived health change (range: -4–4) decrease of 0.21, and for exercise change (range: -4–4) a decrease of 0.31. When comparing individuals who performed 2 HPA with those who performed 4–6 HPA, the average SHAP-based change was -0.10 for weight, 0.09 for perceived health, and 0.17 for exercise change for those with 4–6 HPA.

**Figure 2:**
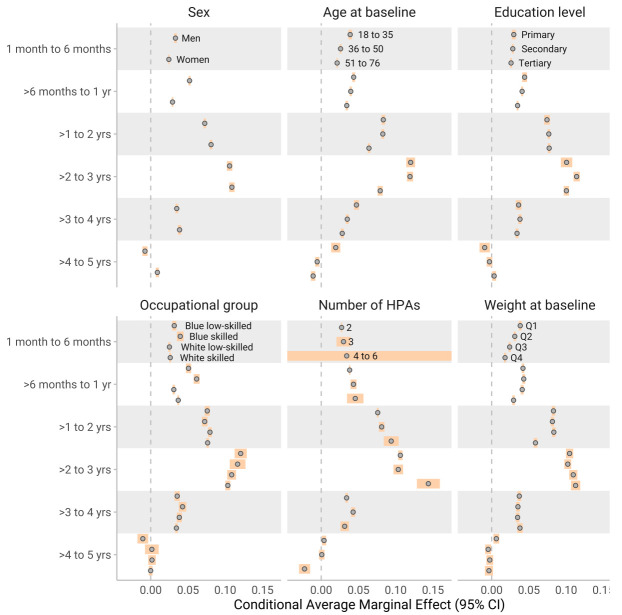
The conditional average marginal effects (AME) of **change in body weight**, and across various demographic and baseline characteristics over different year categories. Each estimate represents the change in perceived health associated with a three-month increment in the time variable, capturing the slope at distinct points in time (similar to how to interpret a regression coefficient). The results are stratified by sex, highest education level, occupation, test counts, age quartiles, and baseline levels of weight. Subgroups with <10 individuals have infinite confidence intervals (CI) to show high uncertainty, while for the rest the confidence intervals were calculated with the t-statistic.

**Figure 3:**
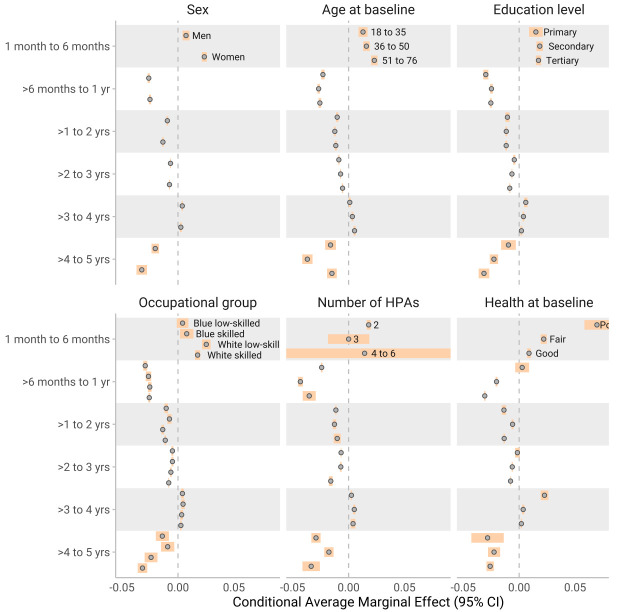
The conditional average marginal effects (AME) of **change in perceived health**, and across various demographic and baseline characteristics over different year categories. Each estimate represents the change in perceived health associated with a three-month increment in the time variable, capturing the slope at distinct points in time (similar to how to interpret a regression coefficient). The results are stratified by sex, highest education level, occupation, test counts, age quartiles, and baseline levels of weight. Subgroups with <10 individuals have infinite confidence intervals (CI) to show high uncertainty, while for the rest the confidence intervals were calculated with the t-statistic.

**Figure 4:**
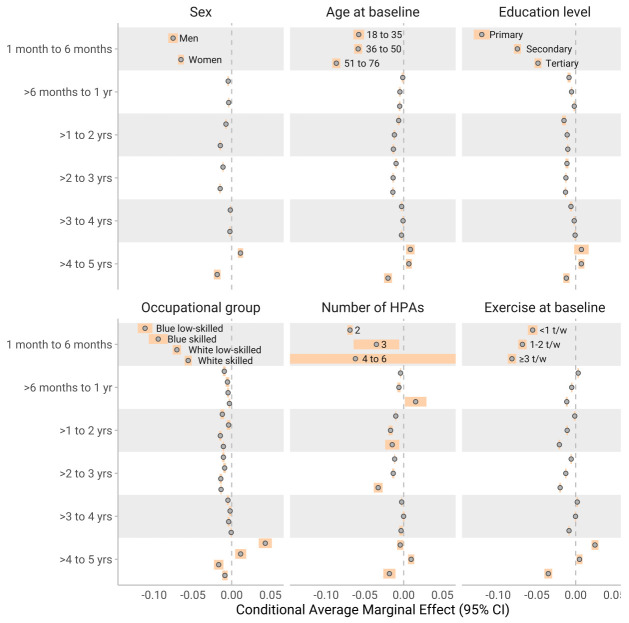
The conditional average marginal effects (AME) of **change in exercise frequency** across various demographic and baseline characteristics over different year categories. Each estimate represents the change in exercise frequency associated with a three-month increment in the time variable, capturing the slope at distinct points in time (similar to how to interpret a regression coefficient). The results are stratified by sex, highest education level, occupation, test counts, age quartiles, and baseline levels of weight. Subgroups with <10 individuals have infinite confidence intervals (CI) to show high uncertainty, while for the rest the confidence intervals were calculated with the t-statistic.

Conditional AME were used to get estimates of subgroups in different time periods. Subgroup results generally paralleled the findings from the SHAP dependency plot (figure 2–4). The most notable differences between subgroups were for exercise frequency, where a higher education level influenced the reduction in slope by 1–6 months positively (a smaller reduction of the slope). During the same period, individuals in occupations requiring higher education exhibited a smaller negative change in exercise outcomes. For similar figures for number of HPA (on y axis) see supplementary figures S3–5.

## Discussion

In the current study including over 100 000 Swedish workers, we investigated how the time interval between the first and last HPA (RQ1), and the number of HPA conducted (RQ2), predict changes in body weight, perceived overall health, and exercise frequency. Another aim was to investigate if the changes differed between subgroups (RQ3). Overall, all three models (outcomes: weight, perceived health, and exercise frequency) demonstrated relatively low predictive performance, characterized by relatively high RMSE and MAE values and low *R*^2^ values. This finding indicates that the variability in changes in weight, perceived health, and exercise frequency was not strongly captured by the included predictors (age at baseline, education, occupational group, number of HPA, time between first and last HPA, and baseline values of the outcome). Nevertheless, the results indicate that a longer duration (range: 0–5 years) between the first and last HPA is associated with a greater increase in body weight compared to the average change. Conversely, individuals who underwent a higher number of HPA had a slight decrease in predicted weight change compared to the average change. Additionally, predicted perceived health and exercise frequency change generally declined as the time between HPA increased, with exercise showing a sharp decline within the first six months. However, an increased number of HPA performed was linked to modest improvements in both health perceptions and exercise frequency.

While the primary trends (RQ1) were consistent across most subgroups, some differences emerged, particularly for weight change where there were consistent patterns of larger increase in weight in the younger age groups (RQ3). For health changes, the conditional AME values were generally very small, with baseline values showing the largest differences between subgroups. Exercise frequency demonstrated a clear dose–response relationship during the first 1–6 months across different education level subgroups (RQ3). Specifically, individuals with primary education experienced the largest decrease in exercise frequency during this period. Similar to previous research we noted that baseline values have a relatively strong impact on the outcomes (12). Those with high health and exercise baseline values had a downward trend, whereas those with low baseline values had an upward trend on average. A similar trend was also observed in weight change but to a lesser degree, which partly may be influenced by the range restriction of the Likert scales whereas weight can always increase or decrease.

The weight gain as a function of time mirror findings in other studies (23, 24). Interestingly, the current study demonstrated an inverse association of the number of HPA performed (RQ2). The discrepancy shows that there could be something influencing the difference, such as a protective effect specifically linked to the number of tests performed. There were also similar patterns for health and exercise frequency, which is consistent with previous studies showing that individualized feedback and ongoing monitoring can enhance health behaviors and outcomes (25, 26). Previous research shows that face-to-face interventions focused on behavioral goal-setting and monitoring of the target behavior positively impact participants motivation (25), which in turn positively impacts health behaviors such as physical activity (27, 28). Hence, conducting repeated HPA may contribute to a positive spiral where initial behavior changes positively impacts motivation, which in turn helps to maintain the behavior change. These findings are also in line with studies showing a dose–response relationship between the number of tests and health outcomes (10, 11).

The steep downward slope in the first 1–12 months for health and exercise frequency change and the general decline in perceived health and exercise frequency as the time between HPA increased may reflect the challenges of sustaining health behaviors without ongoing support (RQ1). Conversely, when individuals received multiple HPA (RQ2), a modest improvement in health and exercise frequency was observed. This trend reinforces the notion that more frequent, ongoing interventions may be necessary to achieve meaningful behavior change. These findings align with reviews highlighting the difficulty of maintaining long-term behavior change without continuous support and help to circumvent obstacles to enact the desired behavior (26, 29). Workplaces, however, might be particularly suited for behavior change interventions that involves social support components and focuses on habit formation and maintenance given that colleagues interact daily and therefore can provide easy-access support and reminders. Furthermore, occupational health services could likely play a crucial role in such interventions by providing support, structure, and maintenance at the individual and organizational level, which most often is beyond the existing resources at the workplace.

Subgroup analyses (RQ3) revealed that younger individuals experienced a larger increase in weight compared to older age groups, which may indicate differing lifestyle factors associated with age. This agrees with previous findings suggesting that weight gain is larger among younger versus older adults (30, 31), indicating that workplace health interventions may need to be tailored differently across age groups. For exercise frequency, the less steep decline among those with secondary and tertiary education suggests that higher educational attainment may buffer against reductions in physical activity. This is consistent with studies indicating that education level is positively associated with health-promoting behaviors such as diet and physical activity (31), however, these findings are not in line with findings in previous reviews showing that the effects on physical activity (6) or BMI (7) did not differ across socioeconomic groups. These reviews found that only participants with high compliance improved their physical activity (6), whereas other factors such as an RCT designs, agentic interventions, focusing on high-risk groups, including a counselling component, using more than five sessions, or were offered at the individual level contributed to a reduction in BMI (7). The impact of education and other socioeconomic factors on the effectiveness of workplace health promotion programs remains inconclusive and more research is needed to better understand the potential effects of socioeconomic inequalities in these programs.

The relationship between the time between HPA should be interpreted cautiously due to potential confounders. Individuals with longer intervals between assessments may differ systematically from those with shorter intervals in ways unrelated to the HPA per se. Also, we cannot determine which specific components of the HPA method or aspects of its delivery may have influenced behavior change in the current study. While the HPA is conducted using a systematic and standardized approach, it was neither originally designed nor implemented as a research intervention. Variability in the delivery of the behavior change dialogue, as well as participant-related factors such as low self-efficacy, limited motivation, or low health literacy, may have affected its impact on health and behavior. Moreover, workplace-related factors – such as organizational conditions and culture – may exert an even stronger influence on the effectiveness of such interventions, and all these may have varied over time. These factors are, however, often overlooked in analyses of effectiveness of workplace health promotion research (32).

The current study also has limitations that should be acknowledged. The observational design together with the machine learning approach limits the ability to draw causal conclusions. Further, the reliance on self-reported measures for perceived health and exercise frequency may introduce reporting bias, likely overestimating positive changes due to socially desirable responses. Additionally, selection bias operates at multiple levels: organizations offering HPA may already prioritize health, and individuals voluntarily participating in more than one HPA may be more health-conscious than non-participants even if participants with differing number of tests have similar life-style values at baseline (table 1). Additionally, while we treated each individual’s change as a separate observation, we acknowledge potential temporal dependencies that our approach may not fully capture. Further the study population was predominantly composed of high-skilled white-collar workers. These high-skilled workers may have greater access to HPA and are often better educated with higher incomes, potentially making lifestyle changes more feasible compared to other occupational groups. While we accounted for occupational categories and education level in our analyses, residual confounding from unmeasured socioeconomic factors (such as work schedule flexibility, job-related stress, or access to health-promoting resources) may still limit the generalizability of our findings. The study also has strengths. The machine learning approach handles interactions and non-linear relationships, uncovering patterns that linear models might miss. In addition, standardized data collection and a large, diverse sample enhance reliability and generalizability, while the use of independent training and testing sets minimizes overfitting. Advanced interpretative methods, such as SHAP values and forward marginal effects, further strengthen the study by clarifying the contribution of each predictor.

### Concluding remarks

Infrequent HPA alone did not appear to substantially influence the lifestyle-related factors studied. However, more frequent HPA, coupled with enhanced feedback and support, may yield small improvements in weight, perceived health, and exercise frequency compared to the average change. Future research should explore mechanisms underlying health assessment effectiveness and identify strategies to enhance outcomes across sociodemographic groups. Furthermore, studies that use causal methods for observational studies with different health outcomes together with randomized controlled trials could help establish causal relationships and assess the long-term impact of regular HPA on various health outcomes. We also encourage an integration of knowledge from the field of behavior change where recent findings from large-scale reviews and meta-analysis suggest that interventions targeting habit formation and maintenance, social support, and helping participants to reduce obstacles to the desired behavior seem particularly potent (29). Combining evidence-based approaches with digital tools and adaptive interventions (33), would enhance understanding of when and how to provide optimal support.

## Supplementary material

Supplementary material

## Data Availability

The data that support the findings of this study are available from HPI but restrictions apply to the availability of these data, which were used under license for the current study and so are not publicly available. Interested parties can request access to the data from the authors upon reasonable request and with the permission of HPI. For inquiries regarding data access, please contact HPI at peter.wallin@hpi.se.
